# Success of the Kousso in the Expulsion of Tape-Worm

**Published:** 1851-02

**Authors:** Thomas Smith


*Success of the Kousso in the expulsion of Tape Worm.*

To the Editor of the Lancet.

SIR—I have much pleasure in forwarding for the Lancet the particulars of a successful trial of the kousso.

Mr. B——, residing in Cheapside, a delicate looking young man, had been troubled with tænia for some years, and had taken the usual remedy, turpentine, with partial success, having at times seen parts of the worm only. I obtained a bottle of kousso from my druggist, which my patient took on Sunday morning, the 15th; after waiting two hours, with the aid of Seidlitz powder, the monster was expelled, *téte et col* complete, measuring twenty-one feet. I need not add that my patient was highly delighted at the good effects of the kousso, and has presented me with the largest specimen of a tape worm I have ever seen.

I am, Sir, your obedient servant,

Thomas Smith.

